# Three-Dimensional-Scanning of Pipe Inner Walls Based on Line Laser

**DOI:** 10.3390/s24113554

**Published:** 2024-05-31

**Authors:** Lingyuan Kong, Linqian Ma, Keyuan Wang, Xingshuo Peng, Nan Geng

**Affiliations:** 1 College of Information Engineering, Northwest Agriculture and Forestry University, Xianyang 712100, China; 2022056038@nwafu.edu.cn (L.K.); 2022056083@nwafu.edu.cn (L.M.); keyuanwang@nwafu.edu.cn (K.W.); pxs@nwafu.edu.cn (X.P.); 2 Key Laboratory of Agricultural Internet of Things, Ministry of Agriculture and Rural Affairs, Northwest A&F University, Xianyang 712100, China; 3 Shaanxi Key Laboratory of Agricultural Information Perception and Intelligent Service, Northwest A&F University, Xianyang 712100, China

**Keywords:** structured light, light stripe-center extraction, 3D data acquisition, pipe measurement, image processing

## Abstract

In this study, an innovative laser 3D-scanning technology is proposed to scan pipe inner walls in order to solve the problems of the exorbitant expenses and operational complexities of the current equipment for the 3D data acquisition of the pipe inner wall, and the difficulty of both the efficiency and accuracy of traditional light stripe-center extraction methods. The core of this technology is the monocular-structured light 3D scanner, the image processing strategy based on tracking speckles, and the improved gray barycenter method. The experimental results demonstrate a 52% reduction in the average standard error of the improved gray barycenter method when compared to the traditional gray barycenter method, along with an 83% decrease in the operation time when compared to the Steger method. In addition, the size data of the inner wall of the pipe obtained using this technology is accurate, and the average deviation of the inner diameter and length of the pipe is less than 0.13 mm and 0.41 mm, respectively. In general, it not only reduces the cost, but also ensures high efficiency and high precision, providing a new and efficient method for the 3D data acquisition of the inner wall of the pipe.

## 1. Introduction

Pipe plays an important role in modern production activities, but the defects, damage, and corrosion of the inner wall of the pipe pose a serious threat to its safe use [1,2]. Conventional methods for inspecting pipes have traditionally depended on hands-on measurements, influenced by elements like material distortion due to contact and the proficiency of workers, leading to reduced work efficiency and inaccurate measurements [3]. Furthermore, the growth of an older population is expected to escalate labor expenses, leading to a trend of different solutions in the industrial inspection sector being sought [4].

As scanning technology progresses, the use of scanners for acquiring 3D pipe data has evolved into a proficient, precise, and nondestructive method [5], offering robust technical assistance in the building and upkeep of pipes, including ultrasonic scanners [6], magnetic scanners [7], structured light scanners [8], and capacitive scanners [9]. These technologies accurately gauge the 3D shape and structure of the pipe’s inner wall without direct contact. In contrast to conventional inspection technologies, these methods present notable benefits, including nondestructive detection, enhanced precision, and superior efficiency.

In the realm of 3D shape acquisition, optical-based structured light scanners stand out among diverse technologies for their notable benefits, including high precision, efficiency, adaptability, and affordability [10,11]. The primary components of structured light scanners include a structured light emitter and a camera [12]. After capturing the object’s surface reflection of structured light, the camera, through stereo vision models and image processing methods, swiftly and precisely gathers the object’s 3D shape data for the analysis and evaluation of feature information [13,14]. Due to the low space requirements of monocular-structured light scanning equipment, this is advantageous for scanning in narrow environments, and can easily cope with the space limitations of narrow diameter pipes.

Given the complex manual measurement operation of narrow diameter pipes, the expensive nature of current apparatus for obtaining the 3D inner wall shapes of pipes, and the difficult equilibrium between speed and precision in conventional center extraction methods for light stripe [15], this study introduces a low-cost, efficient, and high-precision 3D-laser-scanning technology for the inner wall of multi-size pipes is introduced, which can accurately obtain the key data of the shape and size of the inner wall of the multi-size pipe. This technology is essential for assessing the health of pipes, identifying potential problems, and planning maintenance.

## 2. Literature Review

The acquisition of the 3D shape of the inner wall of the pipe is an important research direction in the field of pipe inspection. The primary difficulty is found in the exactness of the 3D-scanning devices and in the efficiency and accuracy of the light stripe’s center extraction. A multitude of academics, both domestically and internationally, have engaged in comprehensive studies in this domain, suggesting diverse solutions and pioneering technologies [16].

### 2.1. 3D-Scanning Technology

Owing to its benefits of being non-contact, highly accurate, and rapid, 3D-scanning technology finds extensive applications across diverse sectors [17,18,19]. In the field of pipe inspection, there has been extensive research on pipe 3D scanning. Shang et al. proposed a single-pass inline pipe 3D-reconstruction method using a depth camera array, which achieved good results in terms of accuracy. However, the size of the measuring equipment designed through this method was difficult to adapt to the inner wall scanning of smaller pipes [20]. Bahnsen et al. studied the 3D scanning of pipes using a single forward-looking RGBD camera. The scanning accuracy was obtained through the use of an active infrared projector or through providing suitable lighting conditions, but the study only achieved centimeter-level accuracy, which was not enough to accurately restore the 3D morphology of the pipe [21]. Yang et al. proposed a pipe 3D-reconstruction method using a 3D-active-stereo omnidirectional-vision scanner, which realized the scanning of the inner wall of a multi-size pipe. However, the measuring equipment designed with this method needed to be in contact with the inner wall of the pipe, which would cause damage to the inner wall of the soft material in the measurement process [22]. In view of the limitations of the above methods in the scanning of the pipe inner wall, a new pipe inner wall-scanning technology based on structured light is proposed in this study.

### 2.2. Extraction of Light Stripe Center

In the linear-structured light 3D-scanning technology, the linear-structured light projected by the linear-structured light emitter is reflected by the target object, and the light stripe appears in the image. The accuracy of the 3D morphology acquisition depends largely on the extraction of the center of the light stripe [23,24]. Initial methods for extracting the center of light stripes predominantly involved the extremum method [25], threshold method [26], and skeleton thinning method [27], among others. These methods rely on the structural features of light stripes to extract centers at the pixel level and are readily influenced by noise. The other is similar to the gray barycenter method (GBM) [28], direction template method [29], curve fitting method [30], and Steger method [31], among others, which are used to determine the center of structured light stripes based on their gray scale features. However, these methods are difficult to balance in terms of accuracy, stability, and speed. Consequently, in the past few years, numerous researchers have enhanced traditional methods for extracting the core of light stripes. Cai et al. calculated the normal direction through principal component analysis, followed with determining the sub-pixel’s central point via second-order Taylor expansion. The technology relies on the Gaussian convolution process, leading to high computational durations [32]. Liu et al. suggested an algorithm for extracting centers, which utilized the Hessian matrix and expanding regions; this method is noted for high accuracy and strong resilience, yet it falls short in real-time efficiency with a wide stripe width [33]. Wang et al. obtained the approximate position of the light stripe center by extracting the skeleton, and then calculated the coordinates of the light stripe center along the skeleton normal direction using the weighted gray barycenter method. The algorithm achieved significant improvement in both the efficiency and the extraction effect [34]. However, current methods for extracting the centers of light stripe lack accuracy in extracting the center of the light stripe in regions of large curvature and are prone to breakpoints. Consequently, an algorithm tailored for arc-shaped light stripes is essential, ensuring sub-pixel accuracy and rapid extraction, satisfying the immediate needs of 3D-measurement systems.

### 2.3. Contributions

The 3D-scanning technology of pipe inner walls faces many challenges, including the demand for high efficiency, high precision, flexible operation, and low equipment cost. In response to these challenges, a new method for the automatic 3D scanning of multi-size pipes using monocular-structured light is proposed. This study’s key innovative contributions include the following:

1. This study proposes an image-processing strategy based on tracking speckles to solve the influence of speckle noise in the image on subsequent light stripe center extractions. The strategy consists of speckle aggregation region extraction, weak speckle grayscale enhancement, and accurate speckle recognition. The problem that the traditional filtering method can not remove the speckle completely is solved by the targeted processing of the speckle.
2. Aiming at the morphological characteristics of arc-shaped light stripes, this study improved the gray barycenter method. On the basis of the traditional gray barycenter method, the center point is modified through fitting a Gaussian curve, and the breakpoint problem in the process of light stripe-center extraction is solved with interpolation based on tangent direction guidance.
3. Utilizing a camera, an annular-structured light emitter, and a mobile control system, this study develops and builds an automatic 3D scanner for the inner wall of multi-size pipes, which enhances the cost efficiency, improves the operational adaptability, and achieves non-contact inner wall detection, providing an effective instrument for the accurate detection of the inner wall of the multi-size pipe.

## 3. Methodology

### 3.1. Creation and Assembly of a Monocular Structured Light 3D Scanner

#### 3.1.1. Elements and Operations of the Scanner

**Figure 1:**
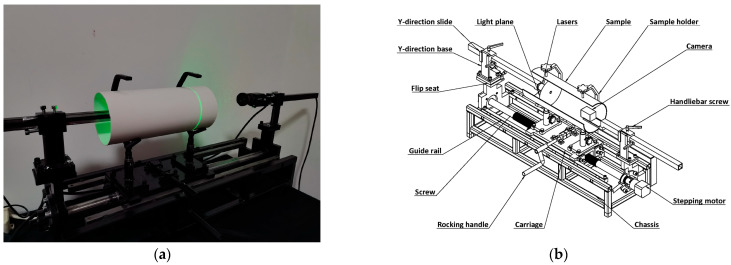
Monocular line-structured light scanner structure. (**a**) Physical diagram. (**b**) Structural diagram.

**Figure 2:**
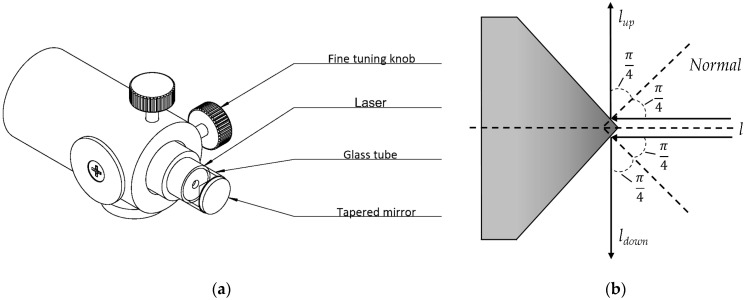
Structure of the annular line-structured light emitter. (**a**) Structural diagram. (**b**) Schematic diagram.

Demonstrated in Figure 1a, this research designed and built a monocular-structured light 3D scanner for the automated 3D scanning of multi-size pipes. Figure 1b illustrates that the primary components of the scanner are an annular-structured light emitter, a camera, and a mobile control platform. As depicted in Figure 2a, the annular-structured light emitter primarily consists of a point laser emitter and a tapered mirror with a 90 degree top angle. As shown in Figure 2b, since the top angle of the tapered mirror is 90 degrees, the angle between the two beams of reflected light $l_{up}$ and $l_{down}$ is

(1) $$\theta =2\cdot \frac{\pi }{4}+2\cdot \frac{\pi }{4}=\pi$$

Consequently, the surface of an annular-structured light can be represented by an arbitrary plane of three degrees of freedom as follows:

(2) ω:Ax+By+Cz+D=0

In order to improve the detection accuracy, the scanner adopts the mode of “sample moving-structured light emitter and camera position fixed”, and is equipped with a stepper motor in order to realize the translation of the sample axis and the control of the image acquisition process, thus ensuring uniform stratification and fast data acquisition. In addition, when the linear-structured light emitter is in a relative position to the camera, different specifications of the pipe can be detected, and the positions of the linear-structured light emitter and the camera are no longer repeatedly adjusted, thus improving the flexibility of the 3D detection of the inner wall of the multi-size pipe.

#### 3.1.2. Adjusting the Annular Structure Light Emitter

**Figure 3:**
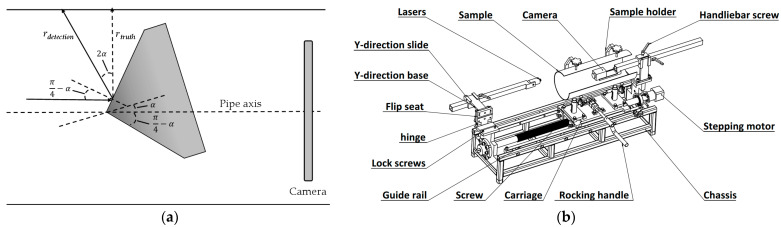
Design of equipment adjustment. (**a**) Analysis of errors generated by annular-structured light emitters. (**b**) Structural diagram in case of flipping.

Installation can lead to a misaligned horizontal alignment between the pipe’s axis and the tapered reflector’s axis, potentially causing errors in the equipment. Figure 3 illustrates that, if the tapered reflector’s axis deviates by an angle value of α from the camera’s optical axis, the resultant light plane strays by an angle value of 2α from the theoretical standard light plane. Consequently, this results in radial inaccuracies as follows:

(3) $$∆r=r_{detection}-r_{truth}=\frac{r_{truth}}{\cos2\alpha }-r_{truth}$$

When the deflection angle is large, it adversely affects the accuracy of the subsequent 3D-shape acquisition. To align the axis of the tapered reflector with the axis of the pipe horizontally, this study uses a correction method based on the light plane equation, aiming to reduce radial errors. In the camera coordinate system, calculate the coordinate deviation value ∆c between the point cloud centroid of each section at different positions of the standard pipe, and adjust the relative position of the sample bracket and the camera according to ∆c, so that ∆c approaches 0, determining the position of the sample bracket and the camera. In this case, the error caused by the deflection angle between the camera optical axis and the pipe axis can be neglected. When the axis of the tapered reflector is parallel to the axis of the pipe, the normal vector n of the light plane in the camera coordinate system is parallel to the z-axis. Therefore, based on the actual parameters of the light plane equation calculated in the camera coordinate system, the annular-structured light emitter is fine-tuned to make the coefficients A and B approach 0, thereby reducing radial errors.

With the adjustment, the relative position relationship between the camera and the annular-structured light emitter can be accurately determined. In addition, by mounting a flip seat at the bottom of the annular-structured light emitter bracket, as shown in Figure 3b, it is possible to ensure that the relative position relationship between the camera and the annular-structured light emitter remains stable after the seat is flipped and reset. This design not only provides convenience for the installation and disassembly of pipes, but also eliminates the need for secondary adjustments when continuously measuring multi-size pipes, thus improving the rigor of the overall operation of the equipment.

### 3.2. Image Processing Strategy Based on Tracking Speckles

The scattering phenomenon caused by the strong reflectivity of the inner wall material will significantly affect the image quality and produce speckle noise near the light stripe. In this study, an image-processing strategy based on tracking speckles is proposed to accurately identify and process the speckle, thus solving the problem that the traditional filtering method cannot completely remove the speckle, and effectively eliminating the influence of speckle noise on the accuracy of the light stripe-center extraction.

#### 3.2.1. Extraction of Speckle Aggregation Regions

Firstly, for very bright scattered speckles, this study employs a bilateral filtering method based on the spatial distribution of the Gaussian filter function [35]. For each pixel $I\left(x,y\right)$ in the image, the value of the pixel after bilateral filtering, $I^{\prime }\left(x,y\right)$, is described as follows:

(4) $$I^{\prime }\left(x,y\right)=\frac{1}{W_{p}}\sum_{s,t\in S}I\left(s,t\right)f\left(\left\|p-q\right\|\right)g\left(\left|I\left(p\right)-I\left(q\right)\right|\right)$$

In the above equation,

(5) $$f\left(\left\|p-q\right\|\right)=e^{-\frac{{\left\|p-q\right\|}^{2}}{2\sigma_{d}^{2}}}$$

and

(6) $$g\left(\left|I\left(p\right)-I\left(q\right)\right|\right)=e^{-\frac{{\left\|I\left(p\right)-I\left(q\right)\right\|}^{2}}{2\sigma_{r}^{2}}}$$

Among them, $p=\left(x,y\right)$ is the current pixel position, $q=\left(s,t\right)$ is a pixel position within the neighborhood S around p, $\sigma_{d}$ is the standard deviation of the spatial kernel, $\sigma_{r}$ is the standard deviation of the range kernel, and $W_{p}$ is a normalization factor.

**Figure 4:**
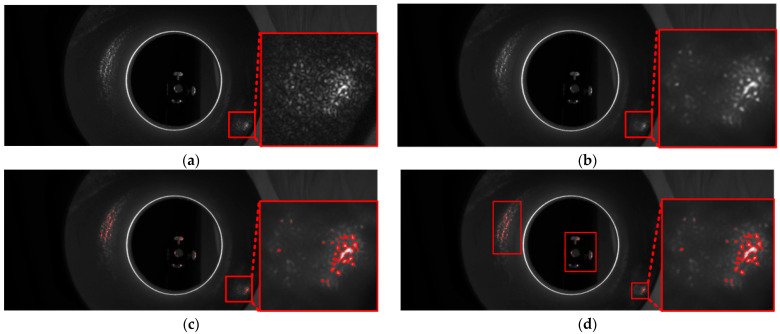
The extraction of initial speckle regions. (**a**) Input image. (**b**) Image after bilateral filtering. (**c**) Initial extraction of speckles. (**d**) Extraction of speckle aggregation regions.

Then, the contour recognition method based on image connectivity analysis [36] is used to extract the connected region in the image, and the connected region whose region is less than the set threshold is determined as the speckle. However, this method can not accurately extract the speckle region, and the phenomenon of less extraction will occur. Therefore, after the initial recognition and extraction of speckles, we further adopted the K-nearest neighbor algorithm to perform cluster analysis on the extracted initial speckles in order to obtain the speckle aggregation region, so as to carry out further speckle recognition against the speckle aggregation region. Figure 4 shows the process of extracting the initial speckle region, where the details of the extraction effect are shown in the red square.

#### 3.2.2. Accurate Extraction of Speckles

Due to the low grayscale of some speckle pixels, this study proposes a new method to enhance weak speckle pixels based on the hyperbolic tangent transform. A morphological operation was performed on the extracted speckle aggregation region, and the mean value avg and standard deviation σ of the morphologically processed images were calculated. The threshold values θ were set as follows:

(7) θ=avg−σ

Each pixel value G(x,y) in the input image I is mapped differently according to its relationship to the threshold θ:

(8) $$G^{\prime }\left(x,y\right)=\left\{\begin{array}{c} N\left({\left(\frac{1}{2}\left(1-tanh\left(\frac{\theta -G\left(x,y\right)}{\theta }\right)\right)\right)}^{k}\right),\;\;G\left(x,y\right)\leq \theta \\ N\left({\left(\frac{1}{2}\left(1+tanh\left(\frac{\theta -G\left(x,y\right)}{\theta }\right)\right)\right)}^{k}\right),\;\;G\left(x,y\right)>\theta \end{array}\right.$$

where $N\left(\cdot \right)$ is a normalization function that normalizes the converted grayscale to a range of $\left[0,255\right]$, $tanh\left(\cdot \right)$ is hyperbolic tangent function, and k is the strength value used for contrast enhancement.

Afterwards, the level set function (LSF) is used to identify the contour of the speckle after the image enhancement. The zero level set of the LSF ϕ(x,y,t) at the temporal variable t is expressed as follows:

(9) $$C=\left\{\left(x,y\right):\phi \left(x,y,t\right)=0\right\}$$

The determination of the contour C is translated into the solution of the partial differential equation (PDE) in Equation (10), that is, the evolution equation of the level set [37].

(10) $$\frac{\partial \phi }{\partial t}=F\left|\nabla \phi \right|$$

where F is the speed function that controls the motion of the contour, and ∇ is the gradient operator. In the traditional level set method, the LSF usually contains irregularity in the evolution process, which leads to numerical errors. While the regularity of the LSF can be maintained through re-initialization as a numerical remedy, it may mistakenly move the set of zero levels away from the intended location. In order to maintain the regularity of the LSF without the need for re-initialization, the distance regular term proposed by Li et al. [38] is used as the new energy term as follows:

(11) $$R\left(\phi \right)≜\int_{\Omega }^{\;}\frac{1}{2}{\left(\left|\nabla \phi \right|-1\right)}^{2}dx$$

where Ω is a domain for the LSF ϕ:Ω→R. Define the energy functional as

(12) $$E\left(\phi \right)=\mu R\left(\phi \right)+E_{ext}\left(\phi \right)$$

where μ>0 is a constant, $E_{ext}\left(\phi \right)$ is the external energy defined by $E_{ext}\left(\phi \right)=\lambda L\left(\phi \right)+\alpha A(\phi )$, and λ>0 and α∈R are the coefficients and the energy functionals $L\left(\phi \right)$ and $A\left(\phi \right)$, which are defined through

(13) $$L\left(\phi \right)≜\int_{\Omega }^{\;}g\delta \left(\phi \right)\left|\nabla \phi \right|dx$$

and

(14) $$A\left(\phi \right)≜\int_{\Omega }^{\;}gH\left(-\phi \right)dx$$

where $g≜1/(1+{\left|\nabla G_{\sigma }*I\right|}^{2})$ is the edge indicator function of image I in the domain Ω, and $G_{\sigma }$ is a Gaussian kernel function with standard deviation σ/δ and H are the Dirac delta function and the Heaviside function [39,40], respectively.

According to the [38], the energy is designed to reach a minimum when the zero-level set of the LSF is at the desired location. According to the variational calculus [41], the $E\left(\phi \right)$ energy can be minimized via calculating the PDE, where $div\left(\cdot \right)$ is the divergence operator.

(15) $$\frac{\partial \phi }{\partial t}=\mu div\left(\frac{\left|\nabla \phi \right|-1}{\left|\nabla \phi \right|}\nabla \phi \right)-\frac{\partial E_{ext}}{\partial \phi }$$

In edge detection in image processing, $E_{ext}$ energy is used to describe the edge information, and the energy functional can be minimized through solving for the following gradient flow:

(16) $$\frac{\partial \phi }{\partial t}=\mu div\left(\frac{\left|\nabla \phi \right|-1}{\left|\nabla \phi \right|}\nabla \phi \right)+\lambda \delta \left(\phi \right)div\left(g\frac{\nabla \phi }{\left|\nabla \phi \right|}\right)+\alpha g\delta \left(\phi \right)$$

**Figure 5:**
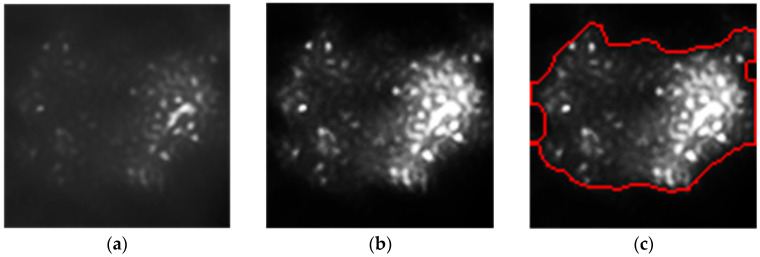
The recognition of speckle region. (**a**) Input image. (**b**) Image enhancement. (**c**) Speckle regions extraction.

Figure 5 shows the process of speckle recognition after image enhancement based on the hyperbolic tangent transformation.

#### 3.2.3. Binarization Processing

In order to suppress the influence of speckles and other background regions, this study first assigned the grayscale of speckle pixels obtained based on Section 3.2.2 to 0, and then set a threshold α to binarize the input image I as follows:

(17) $$g\left(x,y\right)=\left\{\begin{array}{c} I\left(x,y\right),\;\;I\left(x,y\right)\geq \alpha \\ \;\;0,\;\;I\left(x,y\right)<\alpha \end{array}\right.$$

### 3.3. Improved Gray Barycenter Method

In this study, the light stripe region is first divided into four regions, and then the center of the light stripe is extracted for each region, respectively. Finally, the coordinates of the extracted center point of the light stripe are restored to the original image coordinate system. Based on the traditional gray barycenter method, this study is divided into two steps as follows: (1) optimized gray barycenter method via fitting a Gaussian curve, and (2) interpolation guided by the tangent direction.

#### 3.3.1. Optimized Gray Barycenter Method through Fitting Gaussian Curve

In this study, the initial center point of the light stripe is extracted using the traditional gray barycenter method as follows:

(18) $$C_{0}=\frac{\sum xG\left(x,y\right)}{\sum G\left(x,y\right)}$$

**Figure 6:**
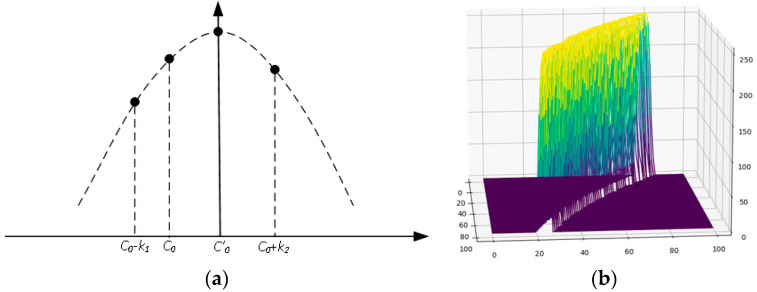
Gaussian curve fitting. (**a**) Gaussian curve calculation. (**b**) The actual grayscale distribution of light stripe.

According to [42], the center line of the light stripe can be quickly and accurately extracted by using the center point of the Gaussian curve as the center point of the light stripe through Gaussian curve fitting. The set of input points is set as $\left\{\left(-n,G\left(C_{0}-n\right)\right),\left(-n+1,G\left(C_{0}-n+1\right)\right),\ldots ,\left(0,G\left(C_{0}\right)\right),\ldots ,\left(n-1,G\left(C_{0}+n-1\right)\right),\left(n,G\left(C_{0}+n\right)\right)\right\}$. As shown in Figure 6a, $C_{0}$ is the initial center point and $G_{0}$ is the grayscale of $C_{0}$. Through the Gaussian curve fitting of the grayscale distribution of $C_{0}$ and the pixels on both sides, the center point ${C\prime }_{0}$ of the corrected light stripe, that is, the central coordinate of the Gaussian curve, is obtained. Equation (19) is the corresponding ideal Gaussian curve function, where a is the amplitude, $C_{0}^{\prime }$ is the central coordinate of the ideal Gaussian curve, and w is the width of the light stripe.

(19) $$G_{i}=ae^{-\frac{{\left(C_{i}-C_{0}^{\prime }\right)}^{2}}{w}}$$

In this study, the least square method is used for the Gaussian curve fitting. In order to construct the error function, the logarithms of both sides of Equation (19) are first taken and converted into polynomials as follows:

(20) $$ln\left(G_{i}\right)=-\frac{C_{i}^{2}}{w}+2\frac{{C_{0}^{\prime }C}_{i}}{w^{2}}+\left(lna-\frac{{C_{0}^{\prime }}^{2}}{w^{2}}\right)$$

Let $ln\left(G_{i}\right)=z_{i}$, $\left(lna-{C_{0}^{\prime }}^{2}/w^{2}\right)=b_{0}$, $2{C_{0}^{\prime }}^{\;}/w^{2}=b_{1}$, $-1/w=b_{2}$, then Equation (20) can be converted to polynomial $z_{i}=b_{0}+b_{1}C_{i}+b_{2}C_{i}^{2}$, and the residual sum of squares is

(21) $$M=\sum_{i=1}^{n}{\left[z_{i}-{(b}_{0}+b_{1}C_{i}+b_{2}C_{i}^{2})\right]}^{2}$$

By minimizing M, $b_{0}$, $b_{1}$, and $b_{2}$ can be solved so that the transverse coordinate of the center point of the corrected light stripe, that is, the central coordinate of the Gaussian curve, can be determined as follows:

(22) $$C_{0}^{\prime }=-\frac{b_{1}}{2b_{2}}$$

The actual grayscale distribution of the light stripe is shown in Figure 6b. Because the Gaussian curve can better simulate the grayscale distribution of light stripe, the center point obtained via Gaussian curve fitting is more accurate than the traditional gray gravity center method, and the interpolation operation will be more accurate and effective in the subsequent processing of light stripe breakpoints.

#### 3.3.2. Interpolation Guided by the Tangent Direction

**Figure 7:**
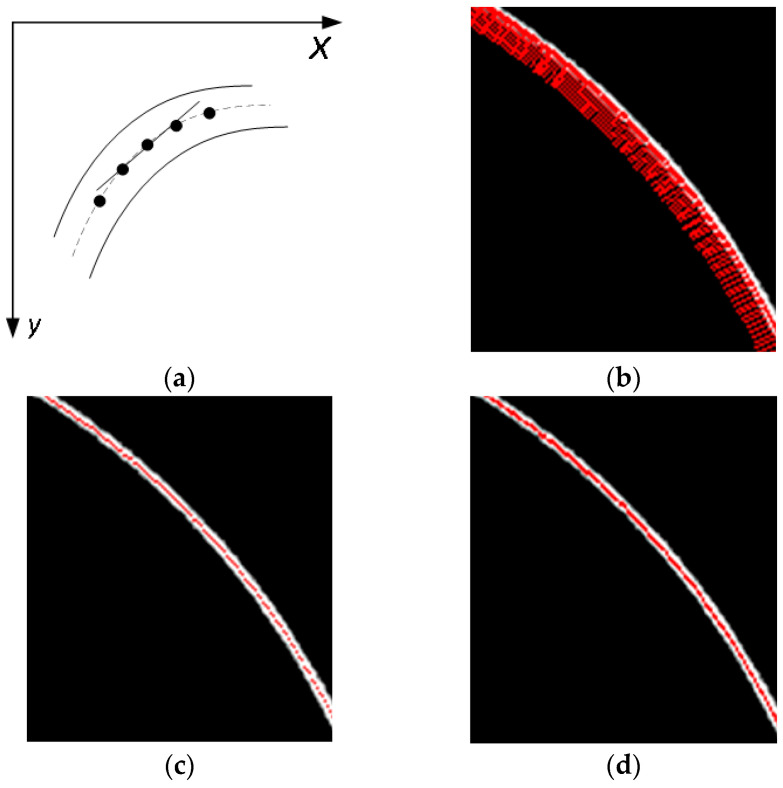
Interpolation point calculation. (**a**) Tangent fitting. (**b**) Center point modified based on Gaussian curve fitting. (**c**) Normal direction. (**d**) The center point after interpolation.

As shown in Figure 7a, through line fitting based on the least square method to the extracted center point of the light stripe and the points on both sides of its neighborhood, the fitted line with equation Ax+By+C=0 can be obtained, and then the slope k of the tangent line of each center point can be extracted. In order to better illustrate the extraction effect, Figure 7b shows the normal corresponding to the tangent line of the center point of a light strip calculated using the above method, and Figure 7c shows the center line of the light stripe after interpolation. According to Equations (23) and (24), the coordinates of the interpolation points along the tangential direction can be calculated to solve the breakpoint problem of the line points in the arc-shaped light stripe, where t is the step length along the tangential direction and α is the angle between the tangential line and the x-axis.

(23) $$\left\{\begin{array}{c} x^{\prime }=x\pm t\cos\alpha \\ y^{\prime }=y\pm t\sin\alpha \end{array}\right.$$

among this,

(24) $$\left\{\begin{array}{c} \cos\alpha =\frac{1}{\sqrt{1+k^{2}}}\\ \sin\alpha =\frac{k}{\sqrt{1+k^{2}}} \end{array}\right.$$

### 3.4. Point Cloud Generation

#### 3.4.1. Monocular Line-Structured Light 3D-Reconstruction Model

**Figure 8:**
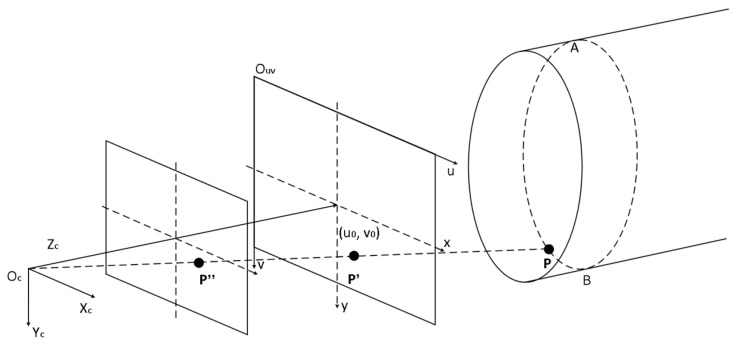
Monocular-structured light 3D-reconstruction model.

This study is based on a monocular line-structured light 3D-measurement model, which obtains its 3D coordinates through the pixel coordinates of the center of the light stripes. As shown in Figure 8, in this model, the light plane PAB, generated by the annular line-structured light emitter, forms circular light stripes on the inner wall of the pipe, where P is a point on the circular light stripe, P′ is the imaging point of P on the imaging plane, and P′′ is the imaging point of P on the normalized plane.

$P(x_{c},y_{c},z_{c})$ is the intersection point between the straight line $O_{c}P\prime$ and the light plane PAB. Since ${P\prime }_{uv}(u,v)$ is known in the pixel coordinate system, the coordinates of this point in the image coordinate system $P^{\prime }(x^{\prime },y^{\prime })$ can be obtained as shown in the following equation:

(25) $$\left\{\begin{array}{c} x^{\prime }=dx\left(u-u_{0}\right)\\ y^{\prime }=dy\left(v-v_{0}\right) \end{array}\right.$$

Among this, $\left(u_{0},v_{0}\right)$ is the coordinate of the origin of the image coordinate system in the pixel coordinate system, dx represents the actual physical size of the unit pixel in the u direction, and dy represents the actual physical size of the unit pixel in the v direction. Since the distance from the center point in the plane to the camera origin is the focal length f, transforming $P^{\prime }(x^{\prime },y^{\prime })$ to the camera coordinate system results in $P^{\prime }(x^{\prime },y^{\prime },f)$. Considering $O_{c}$ as the origin of the camera coordinate system, the equation of the line $O_{c}P\prime$ is as follows:

(26) $$\left\{\begin{array}{c} x_{c}=x^{\prime }t\\ y_{c}=y^{\prime }t\\ z_{c}=ft \end{array}\right.$$

**Figure 9:**
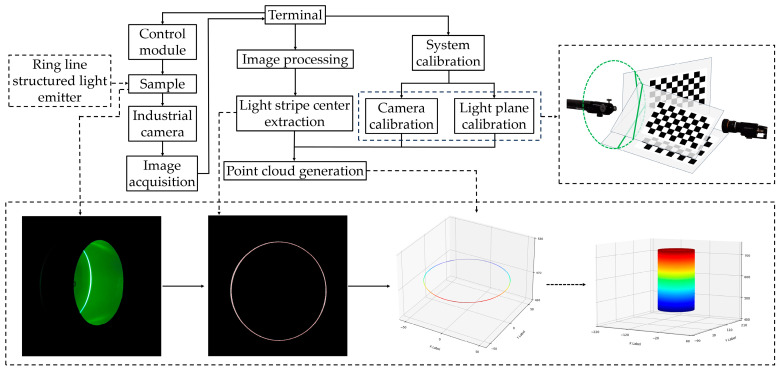
Process of the generating point cloud of the pipe inner wall.

As shown in Figure 9, this study calibrates the camera using the classic Zhang Zhengyou calibration method by adjusting the checkerboard calibration board placed at multiple different angles [43]. Simultaneously calibrating the camera, the light plane generated by the annular line-structured emitter intersects with the blank area of the checkerboard calibration board to form a linear light stripe. The light plane equation is fitted based on the classical least square method via the linear light strips intersected with the calibration plates at different angles. The fitted light plane equation is shown in Equation (2). For ease of calculation, assuming the light plane equation obtained through fitting is as shown in the formula, then

(27) $$z_{c}=ax_{c}+by_{c}+c$$

The coordinates of points $\left(x_{c},y_{c},z_{c}\right)$ on the measured pipe in the camera coordinate system can be obtained from Equations (25)–(27), as shown in the following formula:

(28) $$\left\{\begin{array}{c} x_{c}=\frac{-cdx\left(u-u_{0}\right)}{adx\left(u-u_{0}\right)+bdy\left(v-v_{0}\right)-f}\\ y_{c}=\frac{-cdy\left(v-v_{0}\right)}{adx\left(u-u_{0}\right)+bdy\left(v-v_{0}\right)-f}\\ z_{c}=\frac{-cf}{adx\left(u-u_{0}\right)+bdy\left(v-v_{0}\right)-f} \end{array}\right.$$

Among this, dx, dy, and f are the camera’s own parameters and cannot be calculated only through camera calibration. To enhance the universality of the method, the transformation relationship between the pixel coordinates and camera coordinates is constructed in this study as shown in Equation (29), where $M_{1}$ is the intrinsic parameter matrix obtained through camera calibration.

(29) $$z_{c}\left[\begin{array}{c} u\\ v\\ 1 \end{array}\right]=\left[\begin{array}{ccc} \frac{f}{dx} & 0 & u_{0}\\ 0 & \frac{f}{dy} & v_{0}\\ 0 & 0 & 1 \end{array}\right]\left[\begin{array}{c} x_{c}\\ y_{c}\\ z_{c} \end{array}\right]=M_{1}\left[\begin{array}{c} x_{c}\\ y_{c}\\ z_{c} \end{array}\right]$$

From Equation (29) above, it can be inferred that the coordinates $\left(x_{c},y_{c},z_{c}\right)$ in the camera coordinate system corresponding to a certain pixel (u,v) on the image are as shown in the following equation:

(30) $$\left[\begin{array}{c} x_{c}\\ y_{c}\\ z_{c} \end{array}\right]=z_{c}{M_{1}}^{-1}\left[\begin{array}{c} u\\ v\\ 1 \end{array}\right]$$

To eliminate the influence of variable $z_{c}$ in Equation (30), establish a normalization plane. The image point of P in the image plane and normalized plane is $P^{\prime }$ and $P^{\prime \prime }$, respectively. Because the pixel coordinates of $P^{\prime }$ are (u,v), then the coordinates of P″(x″,y″,1) in the camera coordinate system are as follows:

(31) $$\left[\begin{array}{c} x^{\prime \prime }\\ y^{\prime \prime }\\ 1 \end{array}\right]={M_{1}}^{-1}\left[\begin{array}{c} u\\ v\\ 1 \end{array}\right]$$

$P(x_{c},y_{c},z_{c})$ can be regarded as the intersection point between the line $O_{c}P\prime \prime$ and the light plane PAB. Repeat the above line and surface intersection method to obtain the following coordinates:

(32) $$\left\{\begin{array}{c} x_{c}=\frac{-cx^{\prime \prime }}{ax^{\prime \prime }+by^{\prime \prime }-1}\\ y_{c}=\frac{-cy^{\prime \prime }}{ax^{\prime \prime }+by^{\prime \prime }-1}\\ z_{c}=\frac{-c}{ax^{\prime \prime }+by^{\prime \prime }-1} \end{array}\right.$$

#### 3.4.2. Pipe Inner Wall Reconstruction

As shown in Figure 9, during the scanning process, the pipe is translated along the pipe axis, and the annular-structured light is continuously projected to form a series of circular light stripes at different positions on the inner wall of the pipe. Based on Section 3.4.1, the point cloud data of a single section of the inner wall of the pipe can be obtained using the parameters obtained from the system calibration and the two-dimensional coordinates of the center point of the light stripe [44]. Each of the obtained cross-section point clouds represents the cross-section shape of the inner wall of the pipe at a specific position. Since the pipe axis is parallel to the camera optical axis, the pipe axis is parallel to the z-axis in the camera coordinate system. According to the preset movement parameter k in the scanning stage, the corresponding k units of point clouds in each section are translated along the z-axis, thus achieving a complete point cloud reconstruction of the inner wall of the pipe. The converted coordinates are shown in Equation (33).

(33) $$\left\{\begin{array}{c} {x^{\prime }}_{i}=x_{i}\;\\ {y^{\prime }}_{i}=y_{i\;}\\ {z^{\prime }}_{i}=z_{i}+k \end{array}\right.$$

## 4. Analysis of Results

This study conducts a thorough evaluation of the 3D-laser-scanning technology used to scan the inner wall of the pipe through rigorously designed experiments. The performances of the improved gray barycenter method, traditional gray barycenter method, Steger method, and Wang’s method are compared and analyzed in terms of accuracy and processing speed. Furthermore, it validates the accuracy and stability of the proposed technology through conducting 3D scans and acquiring 3D morphologies of pipes of various sizes. The processing algorithm involved in this study is implemented based on C++ using OpenCV library and PCL library.

### 4.1. Experimental Setup

**Figure 10:**
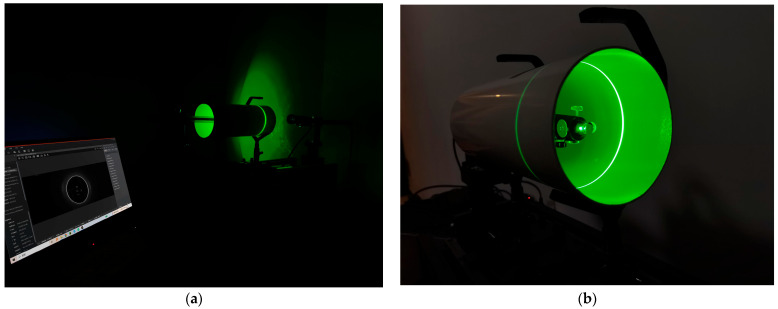
Experimental collection work diagram. (**a**) Overall working diagram. (**b**) Internal workings of the pipe.

In order to avoid the influence of ambient light and brightness saturation on scanning, the experimental collection environment is carried out in a dark room, and the monocular line-structured light 3D scanner is equipped with a 6-megapixel black and white industrial camera and a laser emitter which projects light stripes of 1mm width, as shown in Figure 10. The test pipe is placed on a sample bracket, and an annular-structured light is projected onto the inner wall of the pipe, forming light stripes. During the experiment, the acquisition frame rate is set to eight frames per second, and the acquisition image size is set to 2472×934 pixels. The scanning step of the moving platform is 0.50 mm. The annular-structured light emitter is finely adjusted based on the coefficients of the light plane equation to make the normal vector of the light plane close to parallel with the pipe axis and the camera light axis. The camera calibration results based on Zhang’s calibration method are shown in Table 1. After multiple adjustments, the final fitted light plane equation is Equation (34).

(34) 0.000121087x−0.000227188y−z+472.753=0

### 4.2. Evaluation of the Extraction of the Light Stripe Center

**Figure 11:**
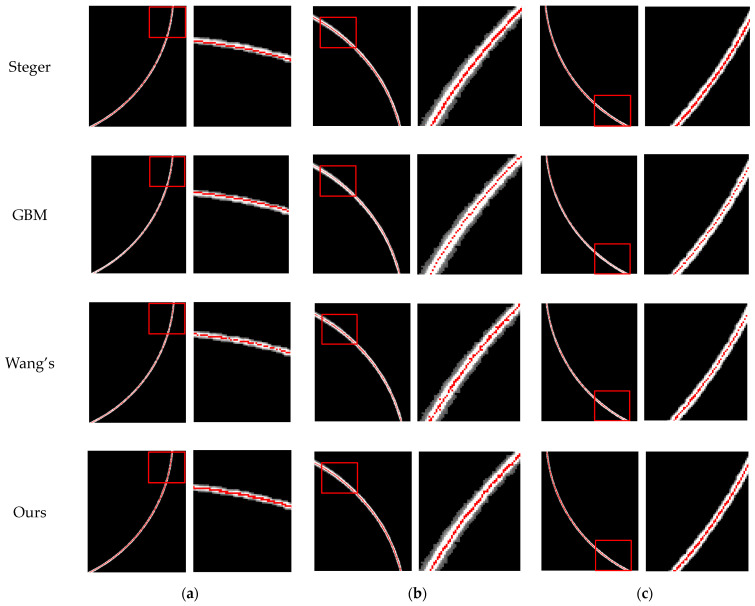
Comparison of the effects in extracting the center of the light stripes [28,31,34]. (**a**) The effect of center extraction in the lower left part of the circular light stripe. (**b**) The effect of center extraction in the upper left part of the circular light stripe. (**c**) The effect of center extraction in the lower right part of the circular light stripe.

In order to evaluate the performance of the improved gray barycenter method, this study conducted comparative experiments with the traditional gray barycenter method, the Steger method, and Wang’s method. Three standard pipes with different inner diameters were selected for the experiments, and images of light stripes at various positions on the inner walls of the pipes were captured to form the experimental dataset, thus ensuring the practical applicability evaluation of the methods. Subsequently, these four methods were applied to extract the centers of the light stripes from the images in the dataset, and the comparative diagrams (as shown in Figure 11) and residual comparison table (as shown in Table 2) of the effects of the four methods in extracting the centers of the light stripes were presented.

From the comparison in Figure 11, it is evident that, although the Steger method can identify the center of the light stripes well, it has poor sensitivity, especially in Figure 11a. Due to the lack of analysis of the geometric properties of the light stripes, the gray barycenter method still has limitations in extracting the centers of light stripes, leading to noticeable discontinuities. Wang’s method can weaken the breakpoint phenomenon by normal weighting, but there are still breakpoints. In contrast, the improved gray barycenter method not only provides more accurate centers of light stripes in arc-shaped regions, but also maintains good continuity. Regarding the runtime, as shown in Table 2, the traditional gray barycenter method takes the least time due to its simple calculations, while the Steger method takes the longest time among all methods due to its involvement in more complex mathematical operations, and Wang’s method ranks in the middle in terms of computing time. Although the improved gray barycenter method increases the computational burden, its running time is still in a low range, second only to the traditional gray barycenter method. This method balances the relationship between efficiency and accuracy, ensures its feasibility and efficiency in practical applications, and has important value in industrial detection and other time-sensitive applications.

### 4.3. Evaluation of the Accuracy of Local Measurements

**Figure 12:**
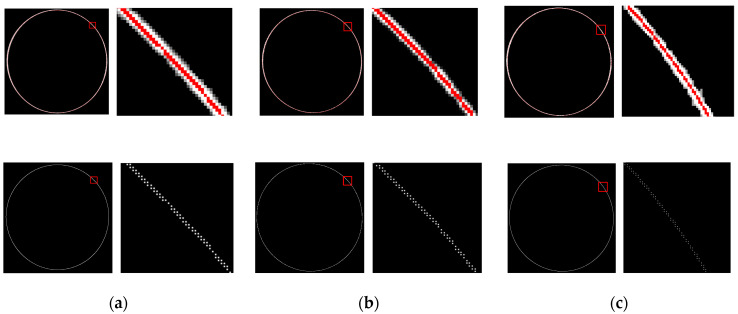
Standard ring gauge measurement diagram. (**a**) Measurement diagram of a standard ring gauge with an inner diameter of 95 mm. (**b**) Measurement diagram of a standard ring gauge with an inner diameter of 105 mm. (**c**) Measurement diagram of a standard ring gauge with an inner diameter of 115 mm.

In order to verify the accuracy of point cloud extraction, a set of standard ring gauges with clear diameters (95 mm, 105 mm, and 115 mm, respectively) were used as the experimental benchmark to verify the local accuracy. As shown in Figure 12, in this experiment, the image captured by the industrial camera was first preprocessed and the center line of the light fringe was accurately extracted. On this basis, the monocular-structured light 3D-reconstruction model in Section 3.4.1 is used to calculate and generate the corresponding point cloud data. As shown in Table 3, after the comparative analysis, it can be found that there is a very small deviation between the measured value and the reference value of the standard ring gauge, that the average deviation is 0.0833 mm, and that the value of RMSE (root mean square error) is also controlled at an excellent level of 0.0839 mm. This result fully proves that the error caused by the coordinate conversion is strictly limited to a very small range, so as to ensure the accuracy and stability of the subsequent 3D-surface-morphology acquisition.

### 4.4. Evaluation of the Accuracy of 3D Surface Morphology Acquisition on Pipe Inner Walls

**Figure 13:**
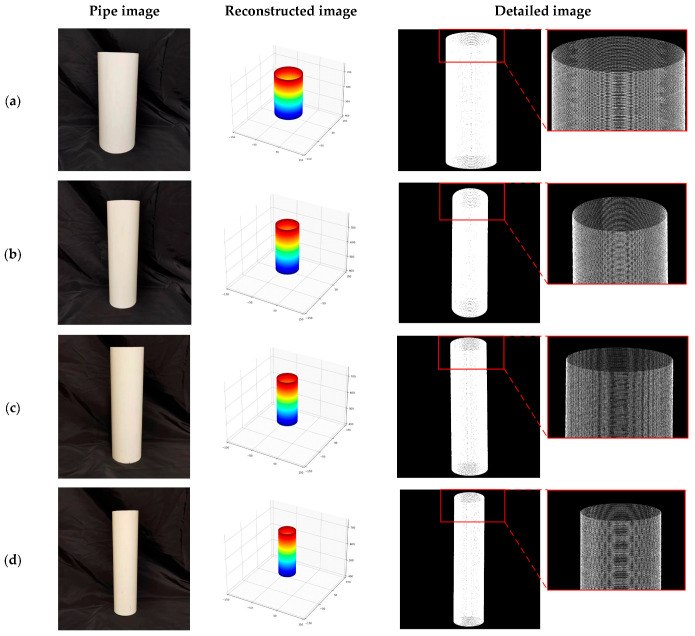
3D-morphology diagram of the inner wall of the pipe. (**a**) 3D-morphology diagram of pipe numbered a. (**b**) 3D-morphology diagram of pipe numbered b. (**c**) 3D-morphology diagram of pipe numbered c. (**d**) 3D-morphology diagram of pipe numbered d.

In this experiment, the inner walls of pipes with four different sizes were scanned to obtain point cloud data of the inner walls, and the reconstructed 3D morphology of the inner walls of the pipes is shown in Figure 13. The point cloud data of the pipe inner walls were processed using a cylindrical dimension analysis method based on cylinder fitting, accurately extracting the inner diameter of the pipes, which were taken as the measured value of the inner diameters of the pipes. Since the program controlled the forward belt of the stepper motor to drive the carriage forward, the gap of all the parts involved in the movement had been compensated and could be ignored, the moving distance was the length change, and the changing size of the moving variable in the program was the length of the pipe. To ensure the accuracy and reliability of the experiment, each pipe was measured five times using a vernier caliper with an accuracy of 0.02 mm, and the average value was taken as the reference value. Subsequently, the experimental measurement values were compared with the reference values in order to evaluate the accuracy of the obtained 3D morphology. The experimental results in Table 4 and Table 5 show that the average deviation between the measured inner diameters of the four pipes and the reference values remain at a low level of less than 0.13 mm, and the RMSE value is 0.1344 mm. Similarly, the average deviation between the measured lengths of the pipes and the reference values remain within an acceptable range of less than 0.41 mm, and the RMSE value is 0.4193 mm, limited by the precision of the screw. High-precision 3D morphology can be obtained for small diameter pipes less than 70 mm. These results indicate that the laser 3D-scanning technology proposed in this study not only applies to the detection of ordinary pipes, but also maintains high accuracy in narrow pipes.

## 5. Conclusions

In order to improve the accuracy and operational flexibility of pipe inspections while reducing the overall costs, this study specifically designed and constructed a monocular line-structured light 3D scanner consisting of a camera, an annular-structured light emitter, and a mobile control platform. Additionally, an image-processing strategy based on tracking speckles is proposed. Through accurate speckle processing, it overcomes the disadvantages of traditional filtering methods, in which it is difficult to completely eliminate speckles, thus reducing the interference of speckle noise on the accuracy of light fringe center positioning. Regarding the morphological characteristics of arc-shaped light stripes, on the basis of the traditional gray barycenter method, the center point is modified via fitting a Gaussian curve, and the breakpoint problem is solved through interpolation based on tangent guidance, which, while ensuring extraction speed, improved the accuracy of the light stripe-center extraction.

In order to evaluate the performance of the proposed laser-based 3D-scanning technology and the improved gray barycenter method, this study utilized a constructed 3D scanner to measure the inner walls of pipes of different sizes. It assessed the accuracy and operation time of the improved gray barycenter method, along with the accuracy of laser-based 3D-scanning technology.

The results show that, when compared with the traditional gray barycenter method, the standard error of light stripe barycenter extraction is reduced by 52%, and, when compared with the Steger method, the average operating time is reduced by 83%. In addition, when compared with Wang’s method, the standard error of optical strip centroid extraction is reduced by 7%, and the average operation time is reduced by 37%. The average deviation of the inner diameter and length of the pipe obtained through laser-3D-scanning technology are less than 0.13 mm and 0.41 mm, respectively, demonstrating sufficient measurement accuracy.

This study provides an efficient and accurate technology for acquiring the 3D morphology of the inner walls of pipes, which is expected to play a significant role in pipe engineering construction and maintenance. However, in future applications, consideration needs to be given to how to optimize the technology to adapt to more complex and harsh pipe environments. This can be achieved through introducing more advanced mechanical designs and control algorithms in order to improve the platform’s motion accuracy and response speed, thus ensuring efficient and stable operation in various environments.

## Figures and Tables

**Table 1:** Results of camera calibration.

Type	Parameter
Internal parameter matrix	$\left[\begin{array}{ccc} 2850.83 & 0 & 1245.61\\ 0 & 2849.61 & 471.12\\ 0 & 0 & 1 \end{array}\right]$
Radial distortion (K_1_, K_2_, K_3_)	[0.1460, −0.8254, 2.7391]
Tangential distortion (P_1_, P_2_)	[−0.0136, −0.0082]

**Table 2:** Performance comparison of different methods for extracting the centers of various light stripes.

Method Type	648 pixel		449 pixel		362 pixel	
	σ/pixel	Time/ms	σ/pixel	Time/ms	σ	Time/ms
Steger [31]	0.40	171.13	0.60	74.02	0.64	50.83
GBM [28]	0.95	17.21	1.33	7.93	1.36	6.21
Wang’s [34]	0.46	41.45	0.68	20.13	0.76	14.22
Ours	0.44	23.62	0.63	12.35	0.68	9.96

**Table 3:** Measurement error results for different standard ring gauges.

Number	(a)	(b)	(c)
Reference/mm	95.00	105.00	115.00
Measurement/mm	94.91	104.91	114.93
Error/mm	0.09	0.09	0.07

**Table 4:** Measurement error results for different pipe inner diameters.

Number	(a)	(b)	(c)	(d)
Reference/mm	106.94	82.66	70.70	60.46
Measurement/mm	106.86	82.54	70.55	60.29
Error/mm	0.08	0.12	0.15	0.17

**Table 5:** Measurement errors of pipe lengths for different pipe sizes.

Number	(a)	(b)	(c)	(d)
Reference/mm	265.28	265.38	263.50	254.98
Measurement/mm	265.00	265.00	263.00	254.50
Error/mm	0.28	0.38	0.50	0.48

## Data Availability

The data presented in this study are available on request from the corresponding author.
